# Boracine (Tetraborate of Soda)

**Published:** 1893-10

**Authors:** 

**Affiliations:** Paris, France


### BORACINE (TETRABORATE OF SODA).[1]

BY DR. DENIS, PARIS, FRANCE.

In one of the last numbers of *L'Odontologie* there appeared a most interesting article, treating of the antiseptic properties of boroborax.

For some time past I have used an identical process and a similar substance called boracine. It is a salt, perfectly purified, resulting from the combination of equal parts of borax and boric acid. This tetraborate of soda is neither caustic, toxic, nor irritating, properties that often belong to antiseptics. Moreover, it has the advantage of being tasteless and odorless, and of dissolving in the proportion of sixteen per cent. I have tested the value of these products, and these are the observations which I have made: First, I have employed it for the disinfection of the dentinal tubuli, and I must say that the results were very satisfactory. However, the results I have obtained have not permitted me to substitute it for the different antiseptics already known. I have also applied it in the treatment of the mucous membrane, in which case it has given me astonishing results.

Among the number of cases, which are too long to enumerate, there are two which are particularly interesting. The first one was upon a rheumatic subject, in the treatment of an abscess of the maxillary sinus, produced in consequence of the too prolonged retention of a first large molar, which was diseased, having caused many consecutive accidents in connection with caries of the fourth degree. I extracted this tooth, and, without entering into details, as is usually the case with an ordinary treatment of such an affection, I treated the abscess, excluding all other antiseptics, and employed only boracine. The second day the suppuration had already begun to diminish, and stopped entirely at the fifth day. That happened three months ago, and my patient has not suffered since. His gums, without being completely healed up, have, however, some time since, regained the normal color. This rapid result has been obtained by putting one coffeespoonful of pulverized boracine in the wound of the sinus, that I took special pains to rinse, before performing the operation, with boracine water of sixteen per cent.; the adding of the powder to the solution has the object of facili-

---

[1] Read before the World's Columbian Dental Congress, Chicago, August, 1893.

tating the absorption of the remedy by the mucus, and in a most direct way, consequently the most efficacious one. The first day I performed three dressings; the second, the third, and the fourth day, two dressings; the fifth day, only one. Every trace of infection has disappeared, and I have often seen my client since that time, enough to convince me entirely that he was thoroughly cured.

The second case is that of a party who, in consequence of a bad extraction of the left cuspid and of the superior incisor, had the anterior part of the maxilla broken in different places; his different teeth were all affected with caries of the fourth degree, which caused many chronic abscesses in the alveolus border and fistulæ in the gums. A month after the extraction of these teeth the patient felt some deep and continuous aching, and the abscess, instead of diminishing, was increasing every day.

It was at that time that he came to consult me to relieve him of his pain. Having detected the presence of sequestra, I cauterized an opening of about one centimetre in width, at the position of the right incisor. In the middle of the principal mass I was lucky enough to extract several deposits of some millimetres, and one measuring exactly a centimetre and a half by one centimetre. As soon as I finished this operation, I rinsed it a number of times with boracine water of sixteen per cent.; then I saturated the wound, so to speak, with some pulverized boracine, and placed a strip of saturated gauze at the orifice in order to prevent the closing of the cavity too quickly, a consequence which would not have permitted ulterior evacuation of the pus, and which also might have prevented my seeing if anything had escaped my investigation. The next day I observed that the abscess had disappeared, and that there remained hardly any trace of suppuration. I performed the same dressing as the day before, without leaving the saturated string. The next day everything was over. Eight days after, the gum had regained, if not entirely its primitive shape, at least its normal color, and no complication had ensued since. This demonstrated, in the first case, collection of considerable pus, which was stopped the fifth day after beginning the treatment; in the other case it was stopped the day after.

I think, therefore, it would be a good thing to generalize the employment, particularly for the buccal mucous membrane, which interests us the most. In order to sustain such results, I could not do better than quote the names of Drs. Galezowski, Landolt, Hubert, La Grange, etc., all of whom now employ boracine in all their clinics with great satisfaction. As a daily antiseptic, its use is plainly indicated against putrefaction of food-particles which remain in the interstices of the teeth, and create an unfavorable element for the multiplication and the station of various microbes, which lodge in the buccal cavity, waiting a favorable opportunity to invade the different organs and produce some injuries. Altogether, I have tried to draw your attention to the employment and the virtues of this new antiseptic, that we salute as having made its entrance into the medical domain.

Believing in the results that it is undoubtedly called to render to our art, owing to its important qualities, it only remains to speak of the properties of this double salt as applied to the enamel of the tooth, and this will be the subject of a new study.

